# Supplementary material for: A Critical View of the Application of the APEX Software (Aqueous Photochemistry of Environmentally-Occurring Xenobiotics) to Predict Photoreaction Kinetics in Surface Freshwaters
Source: Molecules. 2019 Dec 18;25(1):9. doi: 10.3390/molecules25010009 (PMC7017383; doi:10.3390/molecules25010009)
Supplement: Supplementary file 1 [file molecules-25-00009-s001.zip › 3.2.4_gcc-4.4.0.zip!/3.2.4_gcc-4.4.0/3.2.4_gcc-4.4.0/doc/octave/html/interpreter/Matrix-Algebra.html]

Matrix Algebra - Untitled


Next: Function Support,
Previous: Basic Usage,
Up: Diagonal and Permutation Matrices


---

### 20.2 Linear Algebra with Diagonal and Permutation Matrices

As has been already said, diagonal and permutation matrices make it
possible to use efficient algorithms while preserving natural linear
algebra syntax. This section describes in detail the operations that
are treated specially when performed on these special matrix objects.

- Expressions Involving Diagonal Matrices- Expressions Involving Permutation Matrices
